# Response to: “Letter to the editor regarding osteonecrosis of the femoral head in post-COVID-19 patients: a retrospective comparative study”

**DOI:** 10.1186/s13018-025-05930-w

**Published:** 2025-06-05

**Authors:** Jichang Seong, Abduaziz Babakulov, Saodat Asilova, Babamukhamedova Shakhnoza, Makhmudova Nodira, Akbarjon Mirzayev

**Affiliations:** 1 School of Medicine, Central Asian University, Tashkent, 111221 Uzbekistan; 2 Department of Orthopedics and Traumatology, Akfa Medline University Hospital, Tashkent, 100211 Uzbekistan; 3 Department of Orthopedics and Traumatology, Kimyo University Hospital, Tashkent, 100121 Uzbekistan

Dear Editor,

We thank the authors for their interest and thoughtful comments on our recent study exploring the potential association between COVID-19-related factors and osteonecrosis of the femoral head (ONFH) [1]. We also appreciate the opportunity to respond. While many of the points raised by the authors were already addressed in the original article, we are concerned about potential misinterpretations of our study. We provide clarifications on the points raised below.

We acknowledge the limitations inherent to single-center retrospective studies. We would like to clarify that patients with comorbidities such as diabetes mellitus or hypertension were not excluded from our study. However, these conditions were present in a small proportion of our study population, and since they are not COVID-19-specific variables, we did not perform separate analyses on their associations with ONFH. Regarding group size imbalances, we used appropriate non-parametric statistical methods and reported effect sizes to strengthen the validity of our comparisons.

Although we found no significant association between the vaccination status and ONFH, this result should be interpreted with caution due to several limitations in our study. Notably, we lacked detailed information on the type, dose, and timing of vaccines administered, as previously mentioned in the limitations of the original paper [1]. Future studies should account for these confounding factors, as well as potential effects of vitamin intake [2] and genetic predispositions [3].

In response to concerns about steroid dose conversion, our calculations were based on the COVID-19 Treatment Guidelines issued by the National Institutes of Health (NIH), which states that 6 mg of dexamethasone (DEX) is equivalent to 32 mg of methylprednisolone (MPS) [4]. Accordingly, we derived the conversion formula (1 mg MPS = 0.1875 mg DEX) through direct proportional calculation. We also explicitly stated in our limitations that this conversion does not account for the pharmacokinetic and pharmacodynamic differences between the two corticosteroids and was used solely for the purpose of cumulative dose comparison [1].

The notion of “pharmacological synergism” was presented as a hypothesis, not a definitive mechanistic explanation. It was proposed based on the earlier onset of ONFH symptoms observed in the DEX + MPS-treated group compared to the DEX-treated group, and the comparable ONFH symptom onset between Association Research Circulation Osseus (ARCO) stage 2 and stage 3 groups despite significantly higher cumulative steroid exposure in ARCO stage 3 group. If symptom onset were purely dose-dependent, one would expect earlier symptom onset in ARCO stage 3 patients, which was not the case. Thus, we introduced the possibility of a synergistic pharmacological effect, limited specifically to the symptom onset.

We agree that COVID-19 severity and cumulative steroid exposure are often interrelated. To address this, we performed a multivariable logistic regression analysis adjusting for potential confounders, including comorbidities (diabetes mellitus and hypertension), hospitalization durations in both general ward and intensive care unit, COVID-19 lung involvement, and steroid treatment duration. As reported in our results, cumulative steroid dose was the only independent predictor of ONFH severity, as reflected by ARCO stages [1]. We interpret this to mean that variables reflecting COVID-19 severity are likely indirect markers of higher cumulative steroid exposure.

We do not dispute that ONFH is a multifactorial condition. As outlined in the original paper, we acknowledge the potential role of COVID-19-associated endothelial injury and vascular compromise in the development of ONFH. However, Hogea et al. reported that steroid therapy, rather than COVID-19 infection itself, is the primary predictor of avascular necrosis, while also acknowledging the possible impact of the virus on vascular homeostasis and bone metabolism [5]. Similarly, our study identified cumulative steroid dose as the sole predictor of ONFH severity [1].

While magnetic resonance imaging (MRI) is indeed useful tool for definitive diagnosis of ONFH, resource constrains in our region necessitate the use of mixed imaging modalities. Furthermore, it is a common practice to begin the initial assessment with plain radiographs, followed by CT or MRI when necessary, as in fracture cases [6]. Additionally, ARCO staging for ONFH incorporates multiple imaging modalities, including x-ray, computed tomography (CT), and MRI [7]. It is also important to note that identifying subclinical ONFH was beyond the scope of our study.

As stated in the original paper, our recommendation for cautious steroid management is particularly relevant in cases involving combined corticosteroid regimen. Moreover, the safe steroid dosage threshold in the context of post-COVID-19 ONFH remains largely undefined, underscoring the need for further prospective research. Equally important is the future research aimed at establishing standardized treatment protocols, optimizing patient selection for different interventions, and improving long-term outcomes in ONFH management [8–12]. We hope our response clarifies the concerns.

Sincerely,

Jichang Seong

On behalf of the authors.

## Data Availability

No datasets were generated or analysed during the current study.
